# Influence of quality of intensive care on quality of life/return to work in survivors of the acute respiratory distress syndrome: prospective observational patient cohort study (DACAPO)

**DOI:** 10.1186/s12889-020-08943-8

**Published:** 2020-06-05

**Authors:** Christian Apfelbacher, Susanne Brandstetter, Sebastian Blecha, Frank Dodoo-Schittko, Magdalena Brandl, Christian Karagiannidis, Michael Quintel, Stefan Kluge, Christian Putensen, Sven Bercker, Björn Ellger, Thomas Kirschning, Christian Arndt, Patrick Meybohm, Steffen Weber-Carstens, the DACAPO study group, Johannes Bickenbach, Thorben Beeker, Tobias Schürholz, Jessica Pezechk, Jens Schloer, Ulrich Jaschinski, Ilse Kummer, Oliver Kuckein, Steffen Weber-Carstens, Anton Goldmann, Stefan Angermair, Krista Stoycheva, Jörg Brederlau, Nadja Rieckehr, Gabriele Schreiber, Henriette Haennicke, Friedhelm Bach, Immo Gummelt, Silke Haas, Catharina Middeke, Ina Vedder, Marion Klaproth, Michael Adamzik, Jan Karlik, Stefan Martini, Luisa Robitzky, Christian Putensen, Thomas Muders, Ute Lohmer, Rolf Dembinski, Petra Schäffner, Petra Wulff-Werner, Elke Landsiedel-Mechenbier, Daniela Nickoleit-Bitzenberger, Ann-Kathrin Silber, Maximilian Ragaller, Marcello Gama de Abreu, Alin Ulbricht, Linda Reisbach, Kai Zacharowski, Patrick Meybohm, Simone Lindau, Haitham Mutlak, Alexander Hötzel, Johannes Kalbhenn, Christoph Metz, Stefan Haschka, Stefan Rauch, Michael Quintel, Lars-Olav Harnisch, Sophie Baumann, Andrea Kernchen, Sigrun Friesecke, Sebastian Maletzki, Stefan Kluge, Olaf Boenisch, Daniel Frings, Birgit Füllekrug, Nils Jahn, Knut Kampe, Grit Ringeis, Brigitte Singer, Robin Wüstenberg, Jörg Ahrens, Heiner Ruschulte, Andre Gerdes, Matthias Groß, Olaf Wiesner, Aleksandra Bayat-Graw, Thorsten Brenner, Felix Schmitt, Anna Lipinski, Dietrich Henzler, Klaas Eickmeyer, Juliane Krebs, Iris Rodenberg, Heinrich Groesdonk, Kathrin Meiers, Karen Salm, Thomas Volk, Stefan Fischer, Basam Redwan, Martin Schmölz, Kathrin Schumann-Stoiber, Simone Eberl, Gunther Lenz, Thomas von Wernitz-Keibel, Monika Zackel, Frank Bloos, Petra Bloos, Anke Braune, Anja Haucke, Almut Noack, Steffi Kolanos, Heike Kuhnsch, Karina Knuhr-Kohlberg, Markus Gehling, Mathias Haller, Anne Sturm, Jannik Rossenbach, Dirk Schädler, Stefanie D’Aria, Christian Karagiannidis, Stephan Straßmann, Wolfram Windisch, Thorsten Annecke, Holger Herff, Michael Schütz, Sven Bercker, Hannah Reising, Mandy Dathe, Christian Schlegel, Katrin Lichy, Wolfgang Zink, Jana Kötteritzsch, Marc Bodenstein, Susanne Mauff, Peter Straub, Christof Strang, Florian Prätsch, Thomas Hachenberg, Thomas Kirschning, Thomas Friedrich, Dennis Mangold, Christian Arndt, Tilo Koch, Hendrik Haake, Katrin Offermanns, Patrick Friederich, Florian Bingold, Michael Irlbeck, Bernhard Zwissler, Ines Kaufmann, Ralph Bogdanski, Barbara Kapfer, Markus Heim, Günther Edenharter, Björn Ellger, Daniela Bause, Götz Gerresheim, Dorothea Muschner, Michael Christ, Arnim Geise, Martin Beiderlinden, Thorsten Heuter, Alexander Wipfel, Werner Kargl, Marion Harth, Christian Englmeier, Thomas Bein, Sebastian Blecha, Kathrin Thomann-Hackner, Marius Zeder, Markus Stephan, Martin Glaser, Helene Häberle, Hendrik Bracht, Christian Heer, Theresa Mast, Markus Kredel, Ralf Müllenbach, Phillip Sebök, Kathrin Thomann-Hackner, Julika Loss, Bernhard Graf, Michael Leitzmann, Michael Pfeifer, Simon Bein, Vreni Brunnthaler, Carina Forster, Stefanie Hertling, Sophie Höhne, Carolin Schimmele, Elisa Valletta, Philipp Drewitz, Chiara Eberle, Arthur Slutsky, Thomas Bein

**Affiliations:** 1 Institute of Social Medicine and Health Systems Research, Medical Faculty, Otto von Guericke University Magdeburg, Leipziger Str. 44, 39120 Magdeburg, Germany; 2 Medical Sociology, Institute of Epidemiology and Preventive Medicine, University of Regensburg, 93051 Regensburg, Germany; 3 Department of Anesthesia & Operative Intensive Care, University Hospital Regensburg, 93042 Regensburg, Germany; 4 Department of Pneumology and Critical Care Medicine, Cologne-Merheim Hospital, ARDS and ECMO Centre, Kliniken der Stadt Köln, Witten/Herdecke University Hospital, 51109 Cologne, Germany; 5 Department of Anaesthesiology, Emergency and Intensive Care Medicine, University Medicine, 37075 Göttingen, Germany; 6 Department of Intensive Care Medicine, University Medical Centre, Hamburg-Eppendorf, 20246 Hamburg, Germany; 7 Department of Anesthesiology and Operative Intensive Care, University Hospital Bonn, 53127 Bonn, Germany; 8 Department of Anaesthesiology and Intensive Care Medicine, University Hospital Leipzig, 04103 Leipzig, Germany; 9 Department of Anesthesiology and Intensive Care, Klinikum Dortmund, 44137 Dortmund, Germany; 10 Department of Anesthesiology and Intensive Care, University Hospital Mannheim, 68167 Mannheim, Germany; 11 Department of Anesthesiology and Operative Intensive Care, University Hospital Marburg, 35042 Marburg, Germany; 12 Department of Anesthesiology, Intensive Care Medicine, and Pain Therapy, University Hospital Würzburg, 97080 Würzburg, Germany; 13 Department of Anaesthesiology and Intensive Care Medicine, Charité –University Medicine Berlin, 10117 Berlin, Germany

**Keywords:** ARDS, Quality of care, Volume, ICU, Health-related quality of life, Return to work

## Abstract

**Background:**

Significant long-term reduction in health-related quality of life (HRQoL) is often observed in survivors of the acute respiratory distress syndrome (ARDS), and return to work (RtW) is limited. There is a paucity of data regarding the relationship between the quality of care (QoC) in the intensive care unit (ICU) and both HRQoL and RtW in ARDS survivors. Therefore, the aim of our study was to investigate associations between indicators of QoC and HRQoL and RtW in a cohort of survivors of ARDS.

**Methods:**

To determine the influence of QoC on HRQoL and RtW 1 year after ICU-discharge, ARDS patients were recruited into a prospective multi-centre patient cohort study and followed up regularly after discharge. Patients were asked to complete self-report questionnaires on HRQoL (Short Form 12 physical component scale (PCS) and mental component scale (MCS)) and RtW. Indicators of QoC pertaining to volume, structural and process quality, and general characteristics were recorded on ICU level. Associations between QoC indicators and HrQoL and RtW were investigated by multivariable linear and Cox regression modelling, respectively. B values and hazard ratios (HRs) are reported with corresponding 95% confidence intervals (CIs).

**Results:**

877 (of initially 1225 enrolled) people with ARDS formed the DACAPO survivor cohort, 396 were finally followed up to 1 year after discharge. The twelve-month survivors were characterized by a reduced HRQoL with a greater impairment in the physical component (Md 41.2 IQR [34–52]) compared to the mental component (Md 47.3 IQR [33–57]). Overall, 50% of the patients returned to work. The proportion of ventilated ICU patients showed significant negative associations with both 12 months PCS (B = − 11.22, CI −20.71; − 1,74) and RtW (HR = 0,18, CI 0,04;0,80). All other QoC indicators were not significantly related to outcome.

**Conclusions:**

Associations between ICU QoC and long-term HrQoL and RtW were weak and largely non-significant. Residual confounding by case mix, treatment variables before or during ICU stay and variables pertaining to the post intensive care period (e.g. rehabilitation) cannot be ruled out.

**Trial registration:**

Clinicaltrials.govNCT02637011.

(December 22, 2015, retrospectively registered)

## Background

Acute respiratory syndrome (ARDS) is characterised by respiratory failure with either a direct pulmonary (e.g. pneumonia) or extra-pulmonary cause (e.g. sepsis) that requires treatment in intensive care including mechanical ventilation [1]. Approximately 10% of all patients admitted to the ICU develop ARDS [2]. This represents a considerable amount of patients. Further, cost associated with ARDS are very high. For instance, a study from the UK estimated mean societal cost over 1 year including initial ICU treatment cost at £ 44.077 [3]. A US study estimated the median cost due to hospitalization post-discharge at $ 18.756 [4].

With mortality estimated up to 45%, the focus of ARDS research has been on mortality for a long time. With decreasing mortality rates [5] however, the interest in long-term outcomes such as mental health, return to work (RtW) and health-related quality of life (HRQoL) of ARDS patients increased [6–10].

A multi-centre national study in the U.S. showed that nearly half of previously employed ARDS survivors were jobless at 12 months after ARDS and that this was accompanied by substantial lost earnings [11]. If one aims at improving long-term outcome for these patients, it is important to shed light on possible determinants of outcomes such as HRQoL and RtW. We performed a systematic literature review [12] summarising the existing evidence regarding the determinants of HRQoL or RtW in ARDS patients, including 24 highly heterogeneous observational studies. One of the main findings was that the core focus of published research was on clinical and care-related determinants (performance in pulmonary function testing, duration of ICU treatment etc.) which mainly showed small, non-significant effects on HRQoL and RtW. Despite the evident role of the care provided to patients with ARDS in the ICU, surprisingly, the role of quality of care (QoC) for long-term HRQoL and RtW has not been investigated thus far while ARDS mortality has been investigated in relation to university level of care [13].

Against this background, the hypothesis underlying the study presented in this paper was that better QoC (defined by quality indicators) received during the acute ICU stay was associated with better HRQoL and a higher rate of RtW in survivors of ARDS. Thus, our aim was to identify QoC indicators predictive of HRQoL and RtW.

## Methods

### Study design and sample

Methods of the DACAPO (Surviving ARDS: the influence of quality of care and individual patient characteristics on health-related quality of life) study have been described in detail elsewhere [14, 15]. Briefly, adult patients with diagnosed ARDS according to the Berlin definition [16] were recruited in 61 intensive care units (ICUs) across Germany into a cohort study with three postal follow-ups (3, 6 and 12 months). Ethical approval was obtained from the ethics committee of the University of Regensburg (file number: 13–101-0262) and additionally from the ethics committees overseeing the respective study sites.

### Measurements

#### Outcomes

The Short Form-12 self-report questionnaire (SF-12) was used to measure HRQoL [17]. Scores for the Physical Component (PCS-12) and the Mental Component Scales (MCS-12) range from 0 to 100 (higher values indicating better HRQoL). Fifty is the mean value for the general population (German norm values [18]). RtW was captured as self-report, asking whether and when people had returned to their previous or another job (only for persons in employment before admission to ICU). We included data from all participants who returned valid questionnaires up to 13 months after discharge from ICU. Mortality status and date of death were assessed either through reports from the patients’ caregivers or through local population registries.

#### Exposures

QoC was assessed at the level of the participating ICUs once during the period of the study. It was operationalized as structural quality (head of the ICU with additional training in intensive care medicine), process quality (implementation of daily multi-professional ward rounds with documentation of daily therapy goals), volume (number of ventilated patients per year), and general characteristics (membership of the hospital in the ARDS Network Germany). These pre-specified indicators showed limited statistical variance and were thus complemented by additional quality indicators comprising the proportion of physicians with completed specialized ICU training, the availability of weekly microbiological ward rounds, the proportion of ventilated patients on all patients, and general level of care (university hospital versus other hospital). Both the pre-specified and the additional quality indicators were largely based on the published list of German quality indicators in intensive care medicine [14, 19, 20] and together with generally accepted indicators such as volume and level of care have been used in the comprehensive QoC assessment in the DACAPO study.

#### Potentially confounding variables

Variables related to socio-demographic, clinical and care aspects were assessed as potentially confounding variables. The socio-demographic variables included age, sex, living situation (living with versus without a partner), nationality (German, other), and health insurance (statutory, private, other). An education score was derived from information on the participants’ educational and professional levels (according to Lampert et al. [21]). The score ranges between 1 and 7, with higher values indicating higher educational and/or professional achievements. General medical characteristics were captured with regard to body mass index (BMI: kg/m^2^), cause of ARDS (pulmonary / extra-pulmonary), Simplified Acute Physiology Score II (SAPS-II) [22], and Sequential Organ Failure Assessment (SOFA) [23] score, as well as self-reported physician-diagnosed mental disorder before treatment in the ICU (any, none). The Berlin definition of ARDS was modified in terms of the classification of the severity of hypoxemia (PaO_2_/FIO_2_ ratio [P/F ratio]) since the classification into two levels (P/F ratio ≤ 150 mmHg versus > 150 mmHg) may allow better selectivity between mild and severe cases [24, 25].

### Statistical analysis

Owing to the data structure (patients are nested within different ICUs), we used hierarchical linear modeling, testing whether there was systematic variance between the second-level units (ICUs) in the primary outcome at the three follow-ups. For all outcomes, these analyses yielded an intraclass correlation coefficient (ICC) consistently close to zero on the basis of the fully unconditional model and non-significant *p*-values for the likelihood ratio tests indicating non-deterioration in model fit if the random intercept was restricted to zero (data not shown). For this reason, fixed-effects linear, logistic and Cox regression models were applied.

### Outcome: health-related quality of life (HRQoL)

The twelve-month analysis was the primary analysis. For each follow-up (3, 6 and 12 months) and each exposure–outcome combination non-adjusted, minimally adjusted, and fully adjusted linear regression models were used. The minimally adjusted model contained sex, age, and ARDS severity as covariates; all socio-demographic and medical variables which were significantly (*p*-value < 0.05) associated with exposures or outcome at any follow-up period were included in the fully adjusted regression models. Non-standardized regression coefficients with 95% confidence intervals (CIs) were computed as were standardized regression coefficients.

### Outcome: return to work (RtW)

We used Cox proportional hazards models to analyse RtW. The minimally adjusted models had sex, age, and ARDS-severity as covariates. The fully adjusted multivariate Cox models included all covariates that showed significant effects with either outcome or exposure in univariate Cox regression analyses. Observations were censored if RtW did not occur within 395 days following ICU discharge. Hazard ratios (HRs) with 95% CIs were computed.

### Outcome: mortality

We examined the influence of the exposure variables on 1-year mortality in ICU survivors. The set of covariates for the fully adjusted logistic regression models was determined as described for HRQoL and RtW. Odds ratios (ORs) and 95% CIs were computed. A p-value of < 0.05 was considered statistically significant. Analyses were performed using Stata 13.1 (Stata Corporation, College Station, TX, U.S.A.).

## Results

### Descriptive results

**Fig. 1:**
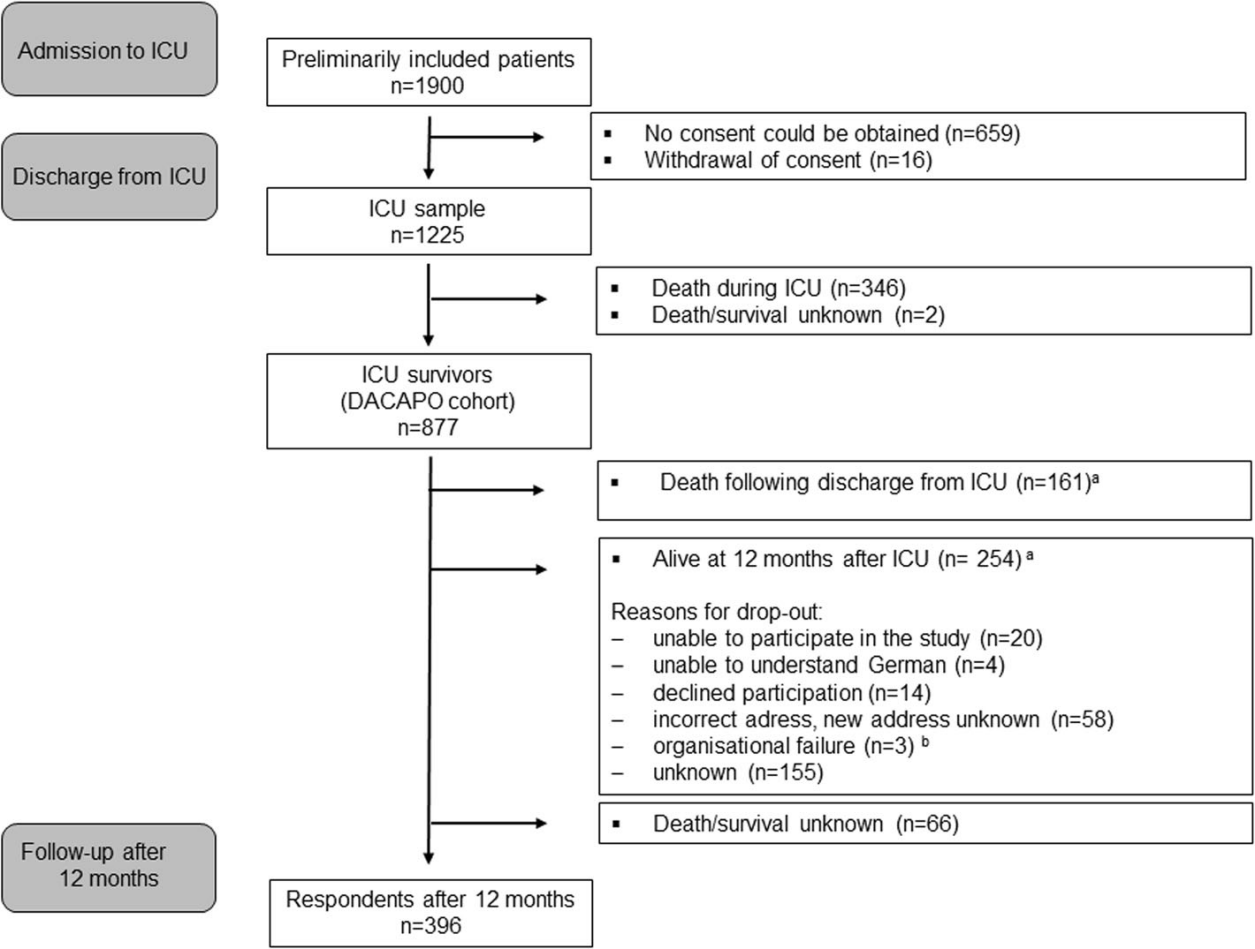
Patient flow. ^a^ For all patients who were lost to follow-up survival was assessed via local municipal population registries. ^b^ Written informed consent and patient data were transferred to the study centre with a delay of more than 12 months; thus, follow-up measurement was not possible within the scheduled follow-up period

We preliminarily included 1900 ARDS patients from 61 ICUs across Germany between September 2014 and April 2016. One thousand two hundred twenty five patients with informed consent formed the initial ICU sample (Fig. 1). Eight hundred seventy-seven patients formed the DACAPO survivor cohort. Four hundred eighty-one patients (54.8%) were lost to follow up for various reasons (e.g.inability to participate). Information on death or survival could not be obtained for 66 patients. At 1 year after discharge, 19.8% had died and 396 persons were followed up.

The socio-demographic and general medical characteristics of the respondents for the twelve-month follow-up are described in an additional file (Table 4, additional file 1). In about 60% the diagnosis was made in a DACAPO ICU, the remainder was transferred. The ARDS was predominantly caused by direct (pulmonary) conditions. The majority of patients (75%) suffered from ‘moderate-to-severe’ ARDS (P/F ratio < 150). A lifetime diagnosis of a mental disorder was reported in 15%.

**Fig. 2:**
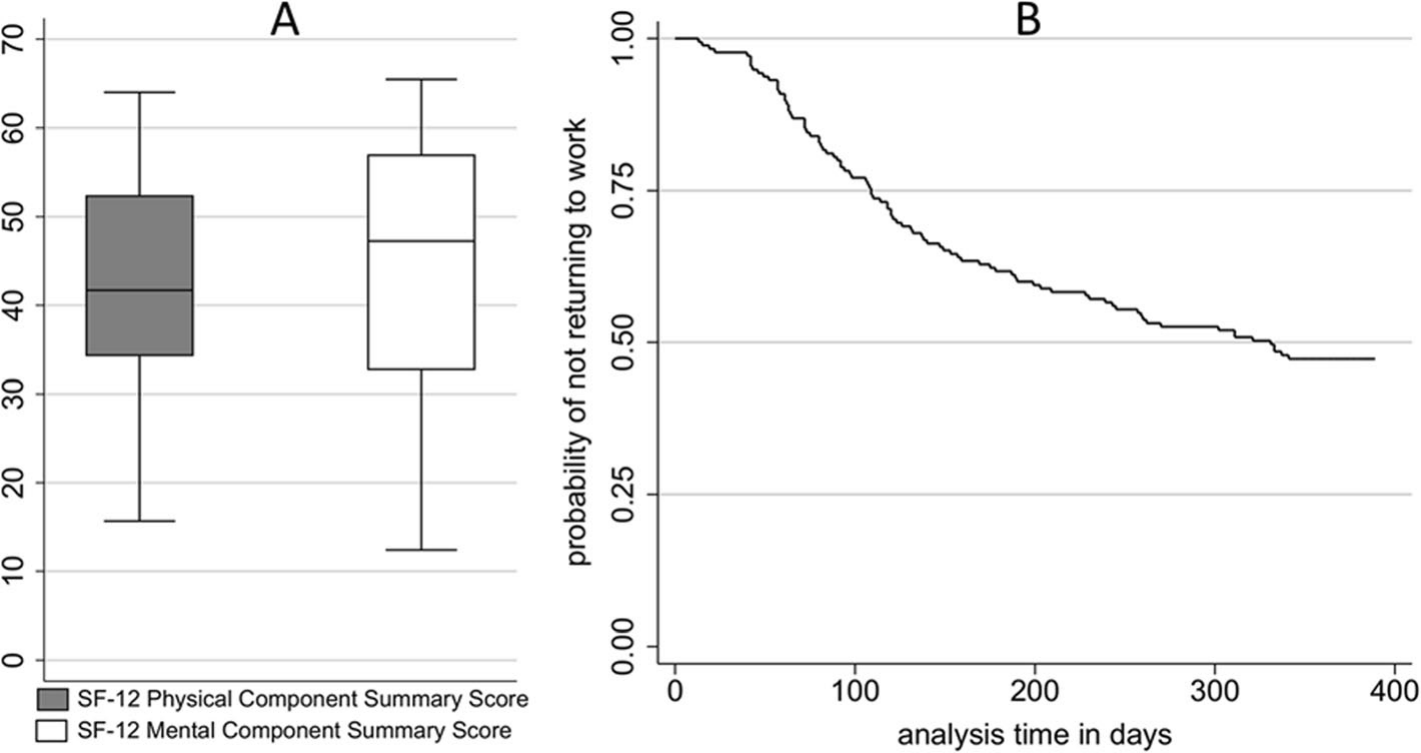
SF-12 values of survivors of ARDS after 12 months (**a**). Probability of not returning to work (**b**)

The general characteristics and the organizational and structural QoC indicators of the participating ICUs were as follows (Table 1): 70% of study centres were members of the German ARDS network and 28 centres were university institutions. The median number of patients ventilated per year was 493 and the median percent of patients who were ventilated was 44%. The twelve-month ARDS survivors reported a median physical SF-12 (SF-12 PCS) of 41 (IQR 35–52) and a mental SF-12 (SF-12 MCS) of 47 (IQR 33–57) Fig. 2. No substantial increase in SF-12 values was observed between 3 and 12 months. Slightly over half of the patients returned to work after 1 year.

**Table 1:** Characteristics of participating ICUs

	ICUs	N
**Volume**		
Number of ventilated patients per year/100, Md (IQR)	48	4.93 (3.87–8.35)
Proportion of ventilated patients on all patients, Md (IQR)	47	0.44 (0.30–0.59)
**Process quality**		
Daily multiprofessional ward rounds with documentation of daily therapy goals, N (%)	51	51 (100)
Weekly microbiological ward rounds, N (%)	52	37 (71.2)
**Structural quality**		
Proportion of physicians with completed specialised training on all physicians, Md (IQR)	52	0.25 (0.16–0.33)
Direction with additional training “intensive medicine”, N (%)	52	51 (98.1)
**General characteristics**		
Member of ARDS-network, N (%)	53	37 (69.8)
Level of care: University hospital, N (%)	53	28 (52.8)

### Analytical results

The main analysis refers to the effect of QoC on HRQoL (Table 5, additional file 2) and RtW (Table 2) after 12 months, and additional analyses were computed for three (Table 6, additional file 3) and 6 months (Table 7, additional file 4) and mortality (Table 3).

**Table 2:** Cox regression analyses of RtW on quality of care in 12 months respondents

12 months follow up: RtW	unadjusted			minimally adjusted ^a^			fully adjusted ^b^		
	N	Haz. Ratio (CI)	p-value	N	Haz. Ratio (CI)	p-value	N	Haz. Ratio (CI)	*p*-value
**Volume**									
N of ventilated patients per year/100	165	0.982 (0.941–1.024)	0.400	138	0.985 (0.940–1.032)	0.525	125	0.975 (0.924–1.029)	0.360
Proportion of ventilated patients on all patients	159	0.366 (0.115–1.163)	0.088	135	0.350 (0.097–1.266)	0.110	122	0.182 (0.041–0.803)	0.024
**Process quality**									
Weekly microbiological ward rounds	175	0.843 (0.485–1.466)	0.546	148	0.743 (0.378–1.459)	0.388	134	0.787 (0.338–1.831)	0.578
**Structural quality**									
Proportion of physicians with completed specialised training on all physicians	175	2.674 (0.459–15.571)	0.274	148	1.230 (0.141–10.726)	0.852	134	4.829 (0.436–53.482)	0.199
**General characteristics**									
Member of ARDS network	175	0.550 (0.311–0.972)	0.040	148	0.614 (0.341–1.106)	0.104	134	0.656 (0.312–1.381)	0.267
Level of Care: University hospital	175	0.829 (0.514–1.339)	0.444	148	0.845 (0.490–1.459)	0.545	134	0.724 (0.392–1.338)	0.303

^a^ adjusted for age, sex, severity of ARDS; ^b^ adjusted for age, sex, severity of ARDS, SOFA score, SAPS-II score, diagnosis of ARDS (participating vs. other ICU), education score

**Table 3:** Logistic regression analyses of mortality on quality of care in ICU survivors

12 months follow up	unadjusted			minimally adjusted ^a^			fully adjusted ^b^		
	N	Odds Ratio (CI)	p-value	N	Odds Ratio (CI)	p-value	N	Odds Ratio (CI)	*p*-value
**Volume**									
N of ventilated patients per year/100	767	1.044 (1.013–1.076)	0.005	660	1.053 (1.017–1.090)	0.004	544	1.048 (1.008–1.090)	0.018
Proportion of ventilated patients on all patients	749	1.476 (0.583–3.737)	0.411	646	1.228 (0.435–3.463)	0.698	533	1.856 (0.558–6.178)	0.313
**Process quality**									
Weekly microbiological ward rounds	811	1.386 (0.869–2.212)	0.171	702	1.483 (0.861–2.552)	0.155	575	1.579 (0.835–2.988)	0.160
**Structural quality**									
Proportion of physicians with completed specialised training on all physicians	811	0.058 (0.009–0.390)	0.003	702	0.072 (0.009–0.604)	0.015	575	0.098 (0.009–1.019)	0.052
**General characteristics**									
Member of ARDS network	814	1.141 (0.645–2.019)	0.651	705	1.269 (0.687–2.342)	0.447	577	1.632 (0.765–3.485)	0.205
Level of Care: University hospital	814	1.636 (1.029–2.602)	0.037	705	1.638 (0.981–2.736)	0.059	577	1.946 (1.059–3.576)	0.032

^a^ adjusted for age, sex, severity of ARDS; ^b^ adjusted for age, sex, severity of ARDS, BMI, cause of ARDS, SAPS-II score, SOFA score, diagnosis of ARDS (participating vs. other ICU), nationality

#### Main analytical results

There was no significant association between most QoC variables and HRQoL at 12 months (Table 5, additional file 2). However, in the fully adjusted analysis, treatment in ICUs that had a higher percentage of ventilated patients was significantly associated with a decreased PCS-12. The analysis of the effects of QoC on RtW after 12 months (Cox regression analyses, Table 2) showed a significantly decreased hazard of RtW for patients treated in institutions with a higher proportion of ventilated of patients but no significant association was found for all further variables.

#### Additional analytical results

In the fully adjusted analysis of the three-month follow-up (Table 6, additional file 3), a higher percentage of patients ventilated was significantly associated with a decreased PCS-12. There was a trend towards a decreased PCS-12 for patients treated in university hospitals (*p* = 0.054). No significant associations were observed for any QoC parameter and 6 months HRQoL (Table 7, additional file 4).

Based on a logistic regression analysis among ICU survivors twelve-month mortality risk (Table 3) was significantly elevated for patients treated in institutions with a higher number of ventilated patients per year. Additionally, patients treated in university hospitals had a significantly increased twelve-month mortality risk (OR 1.946, *p* = 0.032) compared to non-university institutions. A trend was seen for a reduced mortality risk in patients treated in ICUs with a higher percentage of ICU specialist physicians.

## Discussion

### Key findings

The main finding of our study was that of all the quality indicators investigated – taking important confounding factors into account – *only* the proportion of ventilated patients on all patients showed a significant (negative) association with both twelve-months physical HRQoL (PCS-12) and RtW. Secondary findings were a negative significant association of the proportion of ventilated patients on all patients and positive significant associations between the number of ventilated patients per year and university hospital level of care with post ICU mortality risk. No other quality indicator was significantly associated with the outcomes of interest. The DACAPO survivor cohort was characterized by a reduced HRQoL compared to the general population, with a greater impairment in the physical component compared to the mental component. 50% of the surviving patients who were in employment before ARDS had returned to work one year after transferred from the ICU.

### Interpretation, in relation to literature

Our results need careful interpretation. A study by Raymondos et al. [13] showed that the hospital mortality risk of ARDS patients was considerably higher in patients who were treated in non-university hospitals compared to university institutions. However, the present study did not demonstrate a similar effect for HRQoL or RtW 1 year after discharge. A systematic review [26] demonstrated that critically ill patients generally benefit from institutions with high volume regarding mortality with more substantial effects in high risk patients, and there was evidence that this relationship is in part mediated by key hospital or ICU organizational factors. We could not confirm this evidence.

It must be noted though that in contrast to mortality, HRQoL is a complex construct containing individual aspects (multiple dimensions, often operationalized as social, somatic and psychological variables [27]. Our above mentioned systematic review [12] demonstrated significant associations with HRQoL after ARDS only for determinants which were closely related to the scales of the HRQoL instruments and which were measured at the same time as HRQoL.

We were unable to consider variables pertaining to the period after discharge although the post ICU period may play an important role in terms of HRQoL. For instance, a prospective one-year follow-up of 126 patients who received prolonged mechanical ventilation by *Unroe* et al. [28] showed that these experienced multiple ‘trajectories’ after their transfer from the ICU, resulting in frequent readmissions or transitions to various healthcare institutions.

It might be argued that we did not find strong associations between our exposures of interest (i.e. parameters of QoC) and the different outcomes because 1) the exposures showed little variability and 2) effects may have been seen if intermediary outcomes such as length of ventilation or prevention of multiorgan failure would have been considered. However, when it became clear that the quality indicators that were chosen initially did not show sufficient variability, we considered further quality indicators which were related to those that were chosen initially. These indicators did indeed show variability. Second, our research interest was precisely to study modifiable institutional-level indicators of quality of care in relation to patient-level outcomes. We are coming from a public health/health care research perspective in which we can improve health or achieve better disease outcomes, even if we do not know the mechanisms linking exposure to outcome. In light of this background, we did not attempt to explore pathways between QIs, HRQoL, RtW or mortality through intermediary outcomes. Treatment-related exposures not assessed on the institutional but on the individual patient level (parameters of the intensity of acute care management and critical events) have been investigated in a separate paper [29].

### Strenghts and limitations

We were successful in the conduct of a prospective patient cohort study with regular follow-ups. Unfortunately, the number of people lost to follow-up was considerable which may have introduced attrition bias. ICU mortality was not even used as outcome in secondary analysis because the recruitment strategies used focused on the survivor cohort as baseline. For instance, only surviving patients or patients with a legal guardian providing informed consent could be included at some sites, while consent by next-of-kin was acceptable at other sites. Any analysis of factors predictive of ICU mortality would therefore be seriously biased.

Although we adjusted for ARDS severity in our minimally adjusted models, and further corrected for SAPS-II and SOFA scores in the fully adjusted regression models, residual confounding may still be present in relation to ARDS severity/case-mix which might explain our findings. Treatment received before or after ICU care was also not corrected for which might have further contributed to residual confounding.

Measuring QoC in the critical care setting is challenging. In a review by *Flaatten* et al. [30] 63 quality indicators (QI) measuring quality of structure, process and outcome of care were identified, which are in use with a large variation between countries and no single QI was common for all. QIs for structure predominantly refer to the qualification and quantity of health care professionals, as well as the number of ventilated patients per year [31] process quality indicators are more complex, numerous and they refer to actual recommendations of guidelines (ventilation strategies, nutrition, transfusion strategy etc.). These indicators must be defined by experts or Delphi rounds and they are difficult to be monitored continuously in clinical practice. Usually QIs for outcome include standardized mortality (ICU, hospital, 60- or 90-days) or the incidence of decubiti [31]. No previous investigation used QIs for an assessment of the association with HRQoL. In our study we had to use established QIs only assuming that they have value to describe sufficiently the effect of care on patient-reported outcomes.

### Future research

Further, in this paper, we only selected some QIs from the full set. One idea for future research might be to use the full set of QIs to develop a scoring system predictive of patient-level outcome. Such an attempt however is beyond the scope of this paper.

Given the importance of next of kin / family during ICU stay [32], it would be interesting to additionally look at patient-family satisfaction and patient-family engagement in future research.

## Conclusions

In conclusion, most indicators of acute QoC were not significantly associated with one-year HRQoL or RtW in ARDS survivors. Post-ICU exposure of ARDS survivors may have attenuated the assumed effects of high-volume care. Overall, we cannot rule out residual confounding by case mix, treatment variables before or during ICU stay and variables pertaining to the post ICU period.

## Supplementary information

**Additional file 1: **Excel File. **Table 4.** Socio-demographic and general medical characteristics of 12 months respondents. ^a^ derived from educational and professional level^22^, considered for regression analyses. SAPS-II score: Simplified Acute Physiology Score II *without Glasgow Coma Scale;* SOFA score: Sequential Organ Failure Assessment; BMI: Body Mass Index. ^b^as assessed at admission at the DACAPO ICU, ^c^ without consideration of Glasgow Coma Scale.

**Additional file 2: **Excel File. **Table 5.** Linear Regression Analyses of Health-related Quality of Life after 12 months on Quality of Care. ^a^ adjusted for age, sex, severity of ARDS; ^b^ adjusted for age, sex, severity of ARDS, BMI, Education score, SAPS-II score, SOFA score, diagnosis of ARDS (participating vs. other ICU), self-reported physician-diagnosed mental disorder before ARDS diagnosis; Notes: ARDS = acute respiratory distress syndrome; MCS-12 = mental component scale of short-form 12 questionnaire; PCS-12 = physical component scale of short-form 12 questionnaire.

**Additional file 3: **Excel File. **Table 6.** Linear Regression Analyses of Health-related Quality of Life after 3 months on Quality of Care. ^a^ adjusted for age, sex, severity of ARDS; ^b^ adjusted for age, sex, severity of ARDS, BMI, Education score, SAPS-II score, SOFA score, diagnosis of ARDS (participating vs. other ICU), self-reported physician-diagnosed mental disorder before ARDS diagnosis Notes: ARDS = acute respiratory distress syndrome; MCS-12 = mental component scale of short-form 12 questionnaire; PCS-12 = physical component scale of short-form 12 questionnaire.

**Additional file 4: **Excel File. **Table 7.** Linear Regression Analyses of Health-related Quality of Life after 6 months on Quality of Care. ^a^ adjusted for age, sex, severity of ARDS; ^b^ adjusted for age, sex, severity of ARDS, BMI, Education score, SAPS-II score, SOFA score, diagnosis of ARDS (participating vs. other ICU), self-reported physician-diagnosed mental disorder before ARDS diagnosis. Notes: ARDS = acute respiratory distress syndrome; MCS-12 = mental component scale of short-form 12 questionnaire; PCS-12 = physical component scale of short-form 12 questionnaire.

## Data Availability

The datasets generated and/or analysed during the current study are not publicly available due to confidentiality of patient data but are available from the corresponding author on reasonable request.

## Abbreviations

ARDS: Acute respiratory distress syndrome
CRF: Case report form
ICU: Intensive care Unit
IQR: Interquartile range
HRQoL: Health-related quality of life
MCS-12: Mental component summary
Md: Median
PCS-12: Physical component summary
PTSD: Post-traumatic stress disorder
QoC: Quality of care
QoL: Quality of life
RtW: Return to work
SAPS-II: Simplified acute physiology score II
SF-12: Short form 12 survey
SOFA: Sequential organ failure assessment
